# Research progress of traditional Chinese medicine regulating intestinal flora in the treatment of hypertension

**DOI:** 10.3389/fphar.2024.1449972

**Published:** 2024-12-09

**Authors:** Wenjun Chen, Longfei Xiao, Wenlong Guo, Hailin Li, Rong Chen, Zhongyu Duan, Qinghua Chen, Qing Lei

**Affiliations:** ^1^ College of Ethnic Medicine, Yunnan University of Chinese Medicine, Kunming, Yunnan, China; ^2^ Yunnan Key Laboratory of Dai and Yi Medicines, Yunnan University of Chinese Medicine, Kunming, Yunnan, China; ^3^ Department of Thoracic Surgery, The Third Affiliated Hospital of Kunming Medical University, Kunming, Yunnan, China

**Keywords:** hypertension, intestinal flora, traditional Chinese medicine (TCM), intestinal flora metabolites, intestinal barrier function

## Abstract

Hypertension is a common disease; however, it is more prevalent in older adults, and its prevalence is increasing in younger populations. Numerous studies have revealed that hypertension and the composition and functionality of the intestinal flora are closely correlated. The balance of the intestinal flora, intestinal barrier integrity, and metabolite content of the intestinal flora play significant roles in the occurrence and progression of hypertension. Therefore, we performed a comprehensive review of Traditional Chinese medicine (TCM) for hypertension, focusing on the role of the intestinal flora to understand the mechanism by which TCM regulates hypertension through its effects on the intestinal flora. We analyzed the findings using the terms “traditional Chinese medicine,” “hypertension,” “high blood pressure,” “blood pressure,” “intestinal flora,” “intestinal barrier function,” “intestinal flora metabolites,” and other keywords from the China National Knowledge Infrastructure, VIP Chinese Science and Technology, Wanfang Data, PubMed, and ScienceDirect databases. We found that TCM treats hypertension by regulating the balance of the intestinal microbiota, increasing the abundance of beneficial bacteria, reducing the abundance of harmful bacteria, improving intestinal barrier function, increasing compact proteins, reducing intestinal permeability, and regulating the content of intestinal flora metabolites. The use of TCM to treat hypertension by regulating the intestinal flora is a promising therapeutic strategy. However, most studies are limited by small sample sizes and there is a lack of large-scale randomized controlled trials. In the future, multi-center controlled clinical trials are needed to verify the efficacy and safety of TCM, optimize therapeutic protocols, and establish a foundation for the standardized and personalized application of TCM in hypertension management.

## 1 Introduction

Hypertension is characterized by a persistent rise in blood vessel pressure, which increases the risk of injury to the heart, brain, kidneys, and other organs (World Health Organization, 2021). Its pathogenesis includes sympathetic nervous system hyperactivity, renin-angiotensin-aldosterone system activation, vascular endothelial dysfunction, insulin resistance, and neurohumoral factor dysregulation (Yang et al., 2023a). Hypertension is a major cause of premature death worldwide (World Health Organization, 2023). In China, approximately 2.7 million people suffer from hypertension, with only 13.8% of patients achieving adequate control (World Health Organization, 2019). Hypertension in the Chinese population is mainly due to unhealthy lifestyles, such as high-salt diets, overweight and obesity, smoking, alcohol consumption, and insufficient physical activity. Moreover, vasospasm and atherosclerosis occur when blood vessel wall elasticity decreases with age. This also causes diminished function of the blood pressure regulation center, which is an important factor in the development of hypertension. The incidence of hypertension is relatively high in individuals with work pressure, high psychological pressure, chronic tension, and anxiety (Wu and Zhou, 2023). Currently, hypertension management in China relies primarily on Western medicine, including the use of diuretics, angiotensin-converting enzyme inhibitors, β-blockers, angiotensin Il (Ang Il) receptor antagonists, and calcium channel blockers (Zhang and Li, 2023). However, this conventional treatment often requires patients to take two or more antihypertensive drugs simultaneously, and long-term use of these drugs causes drug resistance, adverse effects, and an increased risk of cancer (Wang et al., 2023). TCM can be used to treat hypertension by targeting disease symptoms. It can effectively lower blood pressure, improve accompanying symptoms, reduce side effects, and enhance the therapeutic effects of Western medicine when used in combination. This approach helps protect target organs that are easily damaged by hypertension, such as the heart, brain, and kidneys, thereby improving the quality of life of patients and making them suitable for long-term use.

The human gut, which contains more than 100 trillion microbial cells, significantly influences metabolism. Alterations in the gut flora are associated with factors including diet, the environment, and drug use (Illiano et al., 2020). Gut microbes belong to five main groups: Bacteroidetes, Firmicutes, Actinobacteria, Proteobacteria, and Cerrucomicrobia (Tang et al., 2017; Rahman et al., 2022). Scientific studies have revealed an association between gut flora and hypertension. Additionally, the structure of the intestinal flora, intestinal barrier function, and intestinal flora metabolites are closely associated with hypertension.

Therefore, in this study, we analyzed the findings using the terms “traditional Chinese medicine,” “hypertension,” “high blood pressure,” “blood pressure, “intestinal flora, “intestinal barrier function, “intestinal flora metabolites” and other keywords from the China National Knowledge Infrastructure, VIP Chinese Science and Technology, Wanfang Data, PubMed, and ScienceDirect databases. During the literature screening process, 32 eligible studies were ultimately selected from an initial pool of 350 articles. Inclusion criteria required that the studies explored the relationship between TCM and hypertension, specifically focusing on the role of gut microbiota. The studies encompassed TCM monomers,single-flavor TCM,TCM pairs, and TCM compounding, providing data on the effects of TCM interventions on gut microbiota. All selected literature was published in peer-reviewed scientific journals in English or Chinese. Exclusion criteria included studies that did not focus on the impact of TCM on the gut microbiota in hypertensive patients, non-experimental studies (such as reviews, case reports, and opinion articles), studies lacking detailed data on TCM interventions and gut microbiota changes, and studies with data insufficient to evaluate TCM’s effect on blood pressure regulation. We searched relevant literature in the past 10 years to review the mechanism and current research status of regulating intestinal flora using TCM in the treatment of hypertension.

## 2 Relationship between hypertension and gut flora

### 2.1 Relationship between hypertension and intestinal flora

Specific structural changes in the intestinal flora, such as a decrease in beneficial bacteria and an increase in harmful bacteria, may activate signaling pathways associated with blood pressure regulation, thereby affecting blood pressure. Flora diversity and abundance are usually expressed using Chao1, Abundance-based Coverage Estimator, Operational Taxonomic Units, Shannon, and Simpson indices. Fecal transplantation from human donors with hypertension to germ-free mice showed that elevated blood pressure could be transmitted through the gut flora, revealing the direct effect of gut flora on host blood pressure (Li et al., 2017). Firmicutes and Bacteroidetes account for more than 90% of the total bacterial phyla (Zhang et al., 2021), and their ratio (F/B) is a biomarker of intestinal flora imbalance. F/B was significantly higher in patients with hypertension than in healthy individuals (Cai et al., 2023), and the F/B ratio of spontaneously hypertensive rats (SHRs) was 5-fold higher than that of Wistar rats (Yang et al., 2015). Additionally, hypertension is accompanied by a decrease and increase in beneficial and pathogenic flora, respectively. Beneficial bacteria, such as Bifidobacteria and Lactobacilli, which help maintain gut health and immune system balance, are often reduced in the hypertensive population. Probiotic yogurt reduces blood pressure in SHRs by improving the structure of the intestinal flora, increasing intestinal microbial diversity, and increasing the abundance of short-chain fatty acid (SCFA)-producing bacteria and fecal SCFAs levels (Kong et al., 2021). Intestinal pathogens and their metabolites enter the bloodstream through the mesentery, triggering chronic inflammation and vascular endothelial damage, leading to a decrease in vasodilatory factors, an increase in constrictive factors, and peripheral resistance, ultimately leading to an increase in blood pressure (Yang et al., 2023b). Moreover, probiotics can improve inflammation and lower blood pressure. For example, kefir treatment reduced interleukin (IL)-6 and tissue necrotic factor (TNF)-α protein densities and abolished microglial activation in the hypothalamic paraventricular nucleus and rostral ventrolateral medulla of SHRs (de Almeida et al., 2020). Hence, an imbalance in the gut flora changes metabolites, such as SCFAs, which stimulate the production of 5-hydroxytryptamine, which acts on the vagal nerve and vascular system, causing vasoconstriction and affecting cardiac regulatory regions of the brain through the blood-brain barrier. Furthermore, norepinephrine depresses parasympathetic nerves and, together with 5-hydroxytryptamine, increases blood pressure (Zubcevic et al., 2019). Yan and colleagues (Yan et al., 2020) found that a high-salt diet reduced *Bacteroides* and arachidonic acid levels in the gut of Wistar rats and increased gut-derived corticosterone production and serum and intestinal corticosterone levels, thereby promoting elevated blood pressure. Gamma-aminobutyric acid (GABA) is a neurotransmitter produced by *Bacteroides* via the glutamic acid decarboxylase system. GABA salt may reduce hypertension by decreasing endothelial cell dysfunction and M1 polarization. Moreover, GABA is significantly downregulated in high-salt diet-induced hypertensive rats (Otaru et al., 2021; Son et al., 2021). Thus, the intestinal flora may regulate blood pressure through GABA production. In summary, flora imbalance is closely associated with blood pressure regulation mechanisms, involving changes in flora structure, activation of inflammatory pathways, production of neurotransmitters, and changes in hormone levels, which when combined, contribute to blood pressure regulation.

### 2.2 Relationship between hypertension and barrier function of intestinal

The intestinal barrier is the sum of the structure and functions of the intestine that prevents harmful substances, such as bacteria and toxins, from passing through the intestinal mucosa, entering other tissues and organs, and circulating in the body. The intestinal barrier comprises microbial, chemical, physical, and immune barriers. The microbiological barrier comprises the normal intestinal flora of the host, in which beneficial bacteria support biological defenses through antagonism and immune functions. The chemical barrier includes secretions such as gastric acid, mucus, bile, glycoproteins, and enzymes, which are protective. Columnar epithelial cells and intercellular junctions, such as tight junctions, which separate the intestinal lumen from the internal environment and contribute to protection, constitute a physical barrier. The immune barrier comprises intestinal epithelial cells (iECs), intraepithelial lymphoid tissue (ilEL), lymphocytes, Peyer’s patches, mesenteric lymph nodes, and immunoglobulin A (slgA) from plasma cells (Cui et al., 2019). Gut barrier dysfunction is also associated with hypertension, and various factors, such as intestinal flora imbalance, diet, medications, genetic factors, and diseases, can influence the functioning of the intestinal barrier. Under physiological conditions, intestinal barrier function relies on tight junctions between epithelial cells, the mucus layer, and the effective functioning of the mucosal immune system to maintain intestinal homeostasis (Luissint et al., 2016). However, when these tight junctions are disrupted and mucosal defense mechanisms are impaired, intestinal permeability is enhanced, allowing inflammatory mediators, such as bacteria and endotoxins, to escape into the circulation, which further triggers systemic inflammation. This systemic release of inflammatory mediators leads to vascular endothelial dysfunction and inflammation, which promotes persistent hypertension, exacerbates cardiovascular target organ damage, and promotes the development of refractory hypertension (Yang et al., 2023a; Ge et al., 2024). Approximately one-third of the healthy population is salt-sensitive, and salt-sensitive hypertension accounts for more than 50% of patients with hypertension (Bailey and Dhaun, 2024). The absorption of sodium (Na^+^) and potassium (K^+^) associated with hypertension occurs in the upper ileum; however, the intestinal flora may indirectly influence the absorption and metabolism of these nutrients by modulating the permeability of the intestinal epithelium and the activity of sodium and potassium transporter proteins, which in turn influence blood pressure (Li and Ren, 2023).

### 2.3 Relationship between hypertension and gut flora metabolites

Gut flora metabolites, such as SCFAs, trimethylamine N-oxide (TMAO), lipopolysaccharide (LPS), hydrogen sulfide (H_2_S), and bile acids (BAs), are involved in blood pressure regulation (Ge et al., 2024). SCFAs are produced when gut bacteria ferment dietary fiber, primarily in the cecum and distal colon. It primarily comprises carboxylic acids with fewer than six carbon atoms. The most common s produced include acetate, propionate, and butyrate, which account for 95% of the total SCFAs content (Gao et al., 2024). They can lower blood pressure by dilating blood vessels. Fewer bacteria produce SCFAs when the gut flora is imbalanced, leading to the loss of epithelial barrier function, inflammation, and dysfunction of blood pressure regulation, leading to an increase in blood pressure (Felizardo et al., 2019). Furthermore, SCFAs play a pivotal role in the microbiota-gut-brain axis, influencing the integrity of the blood-brain barrier and the functionality of cells within the brain. Notably, acetate can exert antihypertensive effects by modulating microglia and astrocytes and suppressing neuroinflammation and sympathetic nerve output (Yin et al., 2024). Patients with recalcitrant hypertension have lower levels of propionate in their SCFAs than the healthy population (Ward et al., 2022). Propionate significantly inhibits the hypertensive inflammatory response via CD4^+^ T cell expression in mice (Bartolomaeus et al., 2019). Moreover, treatment with oral butyrate or acetate inhibited the increase in the F/B ratio and blood pressure in spontaneously hypertensive rats (Robles-Vera et al., 2020).

TMAO is a metabolite produced by intestinal microorganisms that metabolizes choline and levulinic acid to trimethylamine (TMA), which is subsequently oxidized in the liver by flavin monooxygenase (FMO) (Gao et al., 2024). TMAO negatively affects the cardiovascular system, especially blood pressure regulation. It enhances the vasoconstrictive effects of Ang II, leading to vascular smooth muscle contraction and increased peripheral vascular resistance, thereby increasing blood pressure (Jiang et al., 2021). Second, TMAO triggers oxidative stress and excessive reactive oxygen species (ROS) damage in vascular endothelial cells and impairs the endothelial ability to release nitric oxide (NO), leading to increased vascular stiffness and uncontrolled blood pressure. Additionally, TMAO promotes the accumulation of advanced glycosylation end products (AGEs), activates the receptor for AGEs (RAGE), triggers inflammation and oxidative stress, damages vascular elasticity and function, and contributes to atherosclerosis and increased blood pressure (Jiang and Duan, 2019; Han et al., 2024).

LPS, or endotoxin, is present in the outer membrane of the most abundant bacteria in the intestinal microbiome. When transferred from the gut to the body, gram-negative bacteria induce inflammation and increase intestinal permeability (Verhaar et al., 2020). LPS binds to Toll-like receptor 4 (TLR4), activating inflammatory signaling pathways, which leads to the release of pro-inflammatory factors (TNF-α, IL-6, and IL-1β). These factors impair vascular endothelial function, inhibit NO production, and weaken vasodilatation, which in turn increases blood pressure (Lu et al., 2008; Zusso et al., 2019). Additionally, LPS induces oxidative stress and excessive damage due to reactive oxygen species (ROS), which affect endothelial cells, exacerbates vascular stiffness, and drives the progression of hypertension (Grylls et al., 2021). Moreover, endotoxins also stimulate the development of hypertension. Simultaneously, endotoxins exacerbate hypertension by stimulating the central nervous system and activating sympathetic nerves, leading to vasoconstriction and increased cardiac output (Dai et al., 2023). In summary, endotoxins mainly contribute to hypertension via inflammation and influence the development and progression of hypertension via inflammation, oxidative stress, and sympathetic activation.

H_2_S gas is reductive, has a high concentration in the colon, and is synthesized mainly by intestinal epithelial cells and intestinal flora through enzymatic reactions (Cirino et al., 2023). It promotes vasodilation by activating ATP-sensitive potassium channels in vascular smooth muscle cells, leading to hyperpolarization of the cell membrane and lowering of blood pressure (Kanagy et al., 2017). Additionally, H_2_S promotes the differentiation and proliferation of regulatory T cells (Tregs), attenuates vascular and renal immune inflammation, and inhibits blood pressure elevation through the sulfation of liver kinase B1 (LKB1) (Cui et al., 2020). The treatment of SHRs with sodium hydrosulfide (NaHS) as a donor of H_2_S resulted in a significant reduction in blood pressure compared with that in Wistar rats (Ni et al., 2018).

**FIGURE 1:**
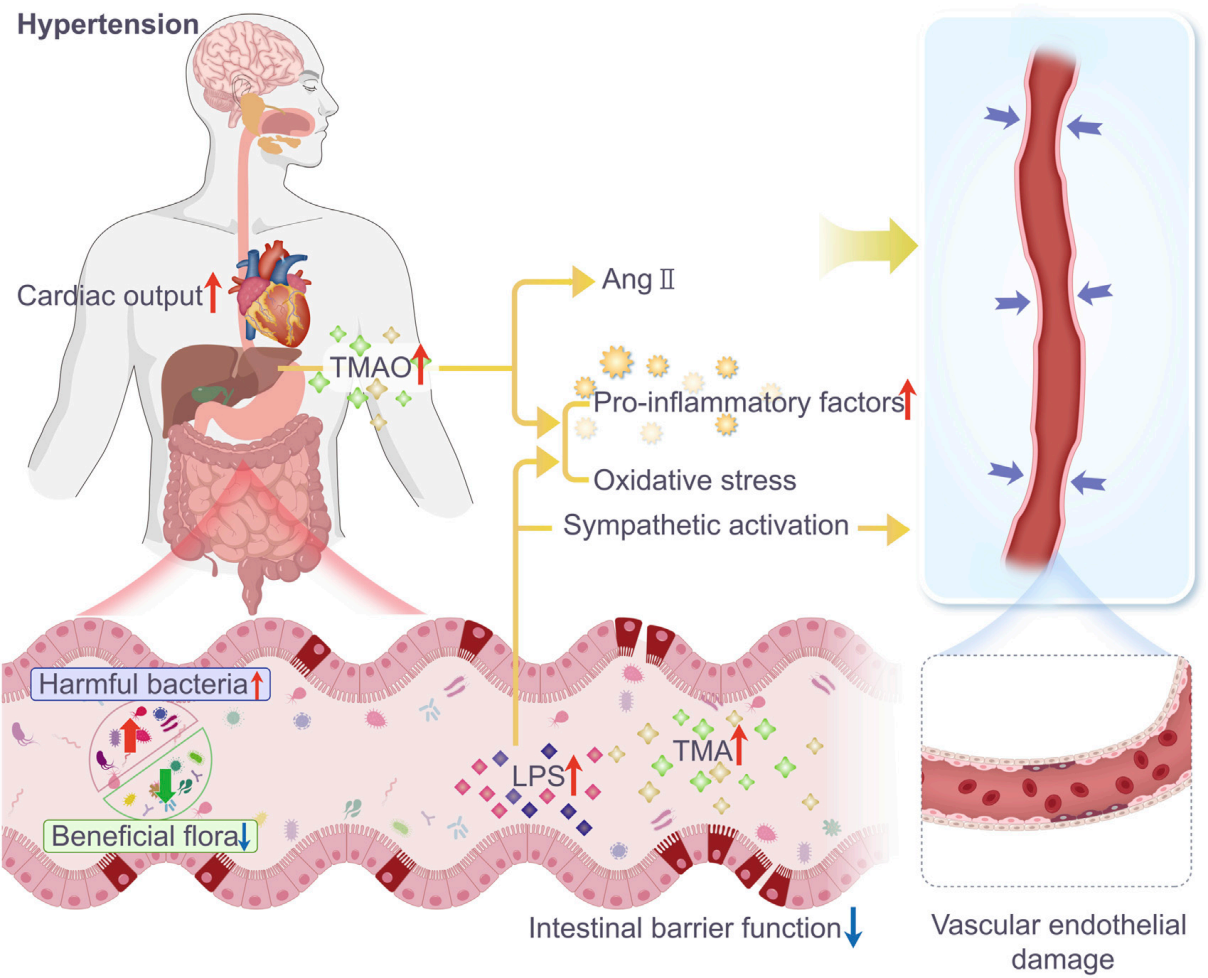
In patients with hypertension, the diversity and abundance of gut microbiota are significantly reduced, and there is a clear dysfunction of the intestinal barrier. The number of beneficial bacteria decreases, while the number of harmful bacteria and gut microbiota metabolites TMAO and LPS increase, leading to a reduction in tight junction proteins in the intestine and increased intestinal permeability. TMAO, a metabolite generated by intestinal microbes from TMA, is oxidized in the liver. It can cause hypertension by enhancing the vasoconstrictive effects of angiotensin II, increasing inflammatory factors, and inducing oxidative stress leading to vasoconstriction. LPS can exacerbate hypertension by releasing pro-inflammatory factors, inducing oxidative stress, and activating the sympathetic nervous system, leading to vasoconstriction and increased cardiac output.

BAs are released into the small intestine during digestion. The intestinal flora further converts them into secondary BAs that promote the absorption of fats and fat-soluble molecules (Tang et al., 2017). BAs lower blood pressure by directly acting on vascular endothelial cells and reducing the vasoconstrictive response induced by norepinephrine. Specific BAs, such as lithocholic acid and taurine goose deoxycholate, promote NO production, which further promotes vasodilation (Tominaga et al., 1988; Guizoni et al., 2020). Additionally, BAs can activate calcium-activated K+ channels (BK(Ca) channels) in patients with hypertension and abnormal calcium metabolism. This condition leads to vasodilation and lowers blood pressure (Ling et al., 2023). BAs also increase vascular smoothness and blood pressure. These acids contribute to lowering blood pressure by activating the farnesylate X receptor (FXR) and G protein-coupled BA receptor in vascular smooth muscle cells and endothelial cells, increasing large-conductance calcium-activated potassium channel activity, and promoting vasodilation (Ishimwe et al., 2022). The intestinal flora influences the host’s metabolic and inflammatory responses by metabolizing BAs and dietary fiber, which may lead to alterations in blood pressure (Fan and Pedersen, 2021). Additionally, BAs may regulate the growth of flora through their antimicrobial effects, safeguarding the structural and functional integrity of the gut and maintaining homeostasis in the intestinal environment (Natividad et al., 2018) (Figure 1).

## 3 Relationship between traditional Chinese medicine, intestinal flora, and hypertension

TCM can treat hypertension by regulating the balance between probiotics and pathogenic bacteria, restoring the balance of intestinal microorganisms, improving intestinal barrier function, and regulating metabolites of the intestinal flora (Yang et al., 2023b). Currently, an increasing number of reports describe how intestinal flora are modified by TCM for treating hypertension, including studies related to TCM monomers, single-flavor TCM, TCM pairs, and TCM combinations. Changes in the intestinal flora in the hypertension model induced by TCM intervention (comparison between the administered and model groups) are shown in Table 1.

**TABLE 1:** Changes in intestinal flora in a model of hypertension induced by TCM intervention (administered group vs model group).

Related Chinese medicine	Type	Intestinal flora	Changes in the diversity index of intestinal flora	Analysis of the metabolite content of the intestinal flora	Sequencing methods	Bibliography
Baicalin	SHR	Akkermansia↑ Allobaculum↑ Bifidobacterium↑ Lachnospiraceae_NK4B4_group↑ Roseburia↑	—	SCFA↑	16S rDNA	Wu et al. (2019)
Baicalin	Hypertensive mice induced by Ang II	g_Alistipes↑ f_Prevotellaceae↑ g_Anaerot r uncu↑ g_Intestinimonas↑ g_Gemmige↑ f_Coriobacteriaceae↑ g_Lachnospiracea_incertea_sedis↑ o_Actinomycetales↑ g_Butyricicoccus ↑ g_Corynebacterium↑	—	—	16S rDNA	Li M. et al. (2022)
Rhynchophylline	SHR	Firmicutes↓ Bacteroidetes↑ Ruminococcus↓ Oscillospira↓ Ruminococcus↓ Prevotella↑	Chao1↑ Ace↑ Shannon↓	—	16S rDNA	Zhang et al. (2023)
Quercetin	SHR	Firmicutes↓ Bacteroidetes↑	Ace↑ Shannon ↑	—	16S rDNA	Zhou et al. (2020)
Resveratrol		Akkermansia↑ Verrucomicrobia↑	—	—	16S rRNA	Chen et al. (2019)
Curcumin	High-salt diet-induced hypertension in mice	*Lactobacillus* murinus ↑	—	—	16S rRNA	Han (2021)
Berberine	Patients with essential hypertension	Firmicutes↓ Lachnospiraceae_NK4A136_group↓ Alistipes↓ Clostridia_UCG_014↓ Ruminococcus↓ *Enterococcus*↓	—	TMAO↓	16S rDNA	Wang et al. (2024)
DOPS	MH rats	Firmicutes ↓ Desulfobacterota↓ Bacteroidetes↑	OTU↑	SCFA↑	16S rRNA	Li B. et al. (2022)
Essential oils (EOs) from Angelica	SHR	Deferribacteres↓ Proteobacteria↓	—	—	16S rDNA	Shen et al., (2020)
Gegen	High-salt diet-induced hypertension in mice	*Clostridium*↑ Lachnospiraceae↓ Anaerotruncus↓ Rhodobacter↓ Eubacteriaceae↓ *Streptococcus*↓	Shannon↑ Simpson ↑		16S rDNA	Li et al. (2020)
Danshen	Hypertensive rats induced by high-salt diet	Prevotellaceae↑	Chao1↑ ACE↑	—	16S rDNA	Qi et al. (2024)
Papaya	SHR	*Bacteroides*↑ Bacteroidaceae↑ Terrisporobacter↑ Peptostreptococcaceae ↑ Firmicutes↓	—	—	16 S rRNA	Chen et al. (2023)
Dendrobium officinale	MH rats	Firmicutes ↓ norank_f__Bacteroidales_S24-7_group↑ Lachnospiraceae↓ Christensenellaceae_R-7_group↓	—	SCFA↑	16S rDNA	Li et al. (2021)
Huangjing	MH rats	*Streptococcus*↑ Desulfobacterota↓ Desulfovibrio↓ unclassified_f_Lachnospiraceae↓ Ruminococcus_torques_group↓ Eubacterium_hallii_group ↓	—	SCFA↑ LPS↓	16 S rRNA	Su et al. (2022)
Duzhong and Cijili	SHR	Actinobacteria↓	PD_whole_tree↑	SCFA↑	16S rDNA	Qi et al. (2019)
Huangqin and Huaihua	SHR	Firmicutes ↓ Bacteroidetes↑ Lactobacillaceae↑ Clostridiales ↓ Bifidobacteriaceae↑	—	—	16 S rRNA	Guan (2020)
Huangqin and Danshen	SHR	*Lactobacillus*↑ Bifidobacterium↑ Akkermansia_muciniphila ↑ *Lactobacillus*↑ *Lactobacillus* reuteri ↑	—	—	16S rDNA	Han et al. (2019)
Sanoshashinto	SHR	*Lactobacillus*↑	—	—	16S rRNA	Wu et al. (2020)
HuanglianJiedu decoction	SHR	*Lactobacillus*↑ Firmicutes↓	Simpson↑	—	16S rDNA	Ma et al. (2020)
Xiaochaihu decoction	Hypertensive patient	*Enterococcus*↓Yeast↓ *Enterobacter*↓ *Bacteroides*↑ *Lactobacillus*↑ Bifidobacter↑	—	—	Japan Mitsuoka contentment method	Wu et al. (2022)
Fufang-Zhenzhu-Tiaozhicapsuledecoction	High-fructose and high-salt (HFS) diet-fed rats	Proteobacteria↑ Verrucomicrobia↑ Fusobacteria↑ Firmicutes↓ *Lactobacillus*↓ Bifidobacterium↓ Burkholderia-Caballeronia-Paraburkholderia↑ Corynebacterium↑ Prevotella↑	—	—	16S rRNA	Chen et al. (2022)
QiangshuJiangya formula	L-NAME induced hypertensive rats	Firmicutes↓ Bacteroidetes↑ Ruminococcus↑	Shannon↑ Simpson↓	—	16S rDNA	Huang (2022)
Erxian decoction	Ovariectomized (OVX) rats	Firmicutes↓ Bacteroidetes↑ Clostridia UCG-014↓ Clostridia vadinBB60 group↓ Ruminococcaceae↓ Muribaculaceae↑ *Bacteroides*↑ Parabacteroides↑ Prevotellaceae NK3B31group↑	—	TMAO↓	16S rRNA	Hu (2023)
TaohongSiwu decoction	High-salt diet-induced hypertension in mice	*Lactobacillus*↑ Allobaculum↑	Chao1 ↓ Shannon↑	BA↑	16 S rRNA	Liu et al. (2023)
Qinggan Yishen Qufeng Compound	Hypertensive mice induced by Ang II	Firmicutes↓ Deferribacteres↓ Acidobacteria↓ Actinobacteria↓ Bacteroidetes↑	Chao1↑ Simpson↑	BA↑	16S rDNA	Zhen (2020)
Zhengan Xifeng decoction	SHR	Proteobacteria ↓ Turicibacter↓ Coprococuus↑ *Clostridium*↑ *Lactobacillus*↓ Ruminococcus↑	OTU↓ Shannon↓ Simpson↑	—	16S rDNA	Yu et al. (2019), Xu et al. (2022)
Chaigui decoction	High-salt diet-induced hypertension in mice	Bacteroidia↑ Clostridia↓	—	—	16S rRNA	Zhu et al. (2023a)
Chaigui decoction	SHR	unclassified_f__S24-7↑	—	—	16S rRNA	Zhu et al. (2023b)
Medicine and food homologous Chinese medicine formula	(2K1C) Hypertension rats	Firmicutes↓ Bacteroidetes↑ *Lactobacillus*↑ Blautia↑ Romboutsia↑ Enterococuus↓	ACE↑ Shannon↑ Simpson ↓	BA↑	16S rDNA	Guo et al. (2023)
Jiawei BanxiaBaizhu Tianma decoction	MH rats	*Streptococcus*↑ Desulfobacter↓ Desulfovibrio↓	—	SCFA↑ LPS↓	16S rRNA	Wu et al. (2024)
Tianma-Gouteng granules	SHR	Desulfovibrio↑, Lachnoclostridium↑ Turicibacter↑ Alluobaculum↓ Monoglobus↓	Shannon ↓ Simpson↑	BA↑	16 S rRNA	Yu et al. (2024)

### 3.1 Traditional Chinese medicine monomers

TCM monomers are purified chemical compounds extracted from TCM and are an important part of the medicinal components of TCM. These monomeric compounds have high purity, well-defined chemical structures, and pharmacological activities, providing strong support for the modernization and development of TCM. Recently, it has been shown that some chemical components of TCM can regulate blood pressure by acting on the intestinal flora. For example, animal experiments have shown that baicalin can significantly inhibit Ang II-induced intestinal epithelial damage and barrier disruption in mice, inhibit inflammatory cell infiltration, and increase the expression of tight junction proteins (Zona Occludens 1 [ZO-1], cingulin, and occludin) and SCFA-producing flora in the intestinal tract (Aliceps and Butyricoccus). Therefore, it enhances intestinal mucosal barrier function, reduces intestinal permeability, protects the structural integrity of the intestine, and lowers blood pressure (Wu et al., 2019; Li B. et al., 2022). Rhynchophylline can optimize the intestinal flora structure by lowering the F/B ratio of SHRs, increasing and decreasing the abundance of beneficial and potentially pathogenic bacteria, respectively, thereby lowering blood pressure (Zhang et al., 2023). Quercetin reduces the F/B ratio, regulates gut flora balance, downregulates the TLR4/NF-κB inflammatory signaling pathway, attenuates myocardial fibrosis, and improves vascular dysfunction and vascular remodeling, thereby lowering blood pressure in SHRs and improving ventricular remodeling (Zhou et al., 2020). Moreover, resveratrol alters the intestinal flora of postnatal adult rats induced using a high-fat diet and NG-nitro-L-arginine-methyl ester. It also decreases the F/B ratio and increases the abundance of beneficial bacteria (Verrucomicrobia and Akkermansia), potentially preventing and reducing hypertension (Chen et al., 2019). Curcumin enhances the abundance of *Lactobacillus muridarum* in the intestines of hypertensive mice fed a high-salt diet. *Lactobacillus muridarum* prevents the exacerbation of salt-sensitive hypertension by regulating helper T cell 17 (TH17) (Han, 2021). Berberine reduces TMA production by modulating the abundance and activity of specific bacteria in the gut microbiota of patients with hypertension. This is achieved by inhibiting CutC/D-containing enzymes, thereby decreasing the plasma levels of TMAO. It also ameliorates vascular endothelial dysfunction by inhibiting the endoplasmic reticulum stress signaling pathway, thus regulating blood pressure (Wang et al., 2024). *Dendrobium officinale* polysaccharide regulates blood pressure by promoting the growth of beneficial flora (*Lactobacillus* and Lachnospiraceae_NK4A136_ group) and decreasing harmful flora (Desulfobacterota and Firmicutes) in the intestine, lowering the F/B ratio, modulating the production of SCFAs, activating the SCFA-GPCR43/41 pathway, improving vascular endothelial function and lipid levels, and enhancing intestinal barrier function. All these functions positively affect hypertension (Li M. et al., 2022). In summary, TCM monomers play a role in lowering blood pressure. This involves regulating the structure and function of the intestinal flora, altering the integrity of the intestinal barrier, decreasing inflammatory responses, and activating metabolite-related signaling pathways of the intestinal flora to reduce the production of harmful metabolites.

### 3.2 Single-flavor traditional Chinese medicines

Many studies have confirmed the efficacy of TCM in preventing and treating hypertension, and its mechanisms of action are closely related to the regulation of intestinal flora. For example, Digupi (Lycii Cortex) reduced the systolic and diastolic blood pressures of SHRs. The groups that received Digupi, including Elusimicrobia, Erysipelotrichia, Erysipelotrichales, Elusimicrobi-Ales, and Muribaculaceae, differed significantly from the model group (Shan et al., 2024). Theolatile oils from Danggui (Angelica sinensis (Oliv.) Diels) were used in SHR. The findings revealed that the abundance of *Aspergillus* spp. in the group that received high-dose Danggui volatile oil, which produces pro-inflammatory toxins, was significantly lower than that in the model group. Therefore, the volatile oil of Danggui may reduce blood pressure by decreasing the abundance of Aspergillus and the production of pro-inflammatory toxins (Shen et al., 2020). Gegen (*Pinus lobata* (Willd.) Ohwi.) and Xiakucao (*Prunella vulgaris* L.) significantly reduced elevated blood pressure induced by a high-salt diet in mice. This may partly restore the diversity of the intestinal flora and increase the abundance of the beneficial bacterium *Clostridium* by elevating the Shannon and Simpson indices. Additionally, it decreased the abundance of Lachnospiraceae, Anaerotruncus, Rhodobacter, Eubacteriaceae, and *Streptococcus*, which are harmful bacteria that are positively associated with hypertension (Li et al., 2020). Danshen (Salvia miltiorrhizage) can regulate blood pressure by improving the diversity and structure of intestinal microorganisms, increasing the abundance of beneficial flora, such as Prevotellaceae, decreasing the F/B ratio, modulating the immune response, and attenuating the inflammatory response and vascular damage induced by a high-salt diet (Qi et al., 2024). Papaya can lower blood pressure in SHRs through its rich dietary fiber content that regulates the intestinal flora, thus lowering the F/B ratio, increasing the activation of G protein-coupled receptor 41 (GPR41) by SCFAs, upregulating the expression of tight junction proteins, and restoring intestinal barrier function by reducing inflammatory factor release (Chen et al., 2023). The ultrafine powder of Dendrobium officinale enhances the gut microbiota and boosts the generation, transfer, and use of SCFAs, subsequently triggering the intestinal-vascular SCFA-GPCR43/41 signaling pathway, which enhances the endothelial function of the blood vessels and ultimately reduces blood pressure in rats with metabolic hypertension (Li et al., 2021). Huangjing (Polygonatum sibiricum Red. Superfine powder, PSP) enhances the integrity of the intestinal barrier by upregulating the expression of tight junction proteins (Claudin-1, occludin, and ZO-1), thereby reducing intestinal permeability and effectively reducing pathogens and harmful substances from LPS in the blood circulation. Additionally, PSP regulates intestinal flora by decreasing and increasing the abundance of harmful (Desulfobacter and Desulfovibrio) and beneficial (*Streptococcus*) bacteria, respectively. The combined effects improve blood pressure in rats with metabolic hypertension (MH) induced by a high-sugar, high-fat, and complex alcohol diet (Su et al., 2022). In summary, single-flavor TCM may exert blood pressure-lowering effects by improving the balance of intestinal flora, enhancing intestinal barrier function, promoting the metabolites produced by beneficial flora such as SCFAs, improving vascular endothelial function, and attenuating inflammatory responses.

### 3.3 Traditional Chinese medicine pairs

Some TCM pairs have been experimentally validated for the treatment of hypertension. Recently, the intestinal flora has received increasing attention as a novel therapeutic target for the treatment of hypertension, and studies on the effects of TCM pairs on intestinal flora have also increased. Duzhong (Eucommia ulmoides Oliv.) and Cijili (Tribulus terrestris) spontaneously reduced the abundance of actinomycetes in older rats with hypertension. The abundance of actinomycetes in these rats increases the level of SCFAs in feces and regulates blood pressure by reducing the production of inflammatory factors (Qi et al., 2019). Huangqin (Scutellaria baicalensis Georgi) and Huaihua (Sophora japonica L.) increased the biodiversity of the intestinal flora in SHRs, decreased the F/B ratio, and increased the abundance of beneficial bacteria. Moreover, Lactobacillaceae and Bifidobacteriaceae ameliorated intestinal damage, repaired intestinal villi, and increased mucin expression, which reduced blood pressure (Guan, 2020). Huangqi (Arabidopsis membranaceus (Fisch.) Bge. var. mongholicus (Bge) Hsiao) and Danshen may increase *Akkermansia muciniphila* by increasing the abundance of probiotics such as *Lactobacillus* spp., Bifidobacterium spp., *Lactobacillus intestinalis*, and *Lactobacillus reuteri*, which regulate the structure and diversity of the intestinal flora and decrease the F/B ratio, improving the intestinal microecology and further reducing the blood pressure of SHRs (Han et al., 2019).

### 3.4 Traditional Chinese medicine compounding

**FIGURE 2:**
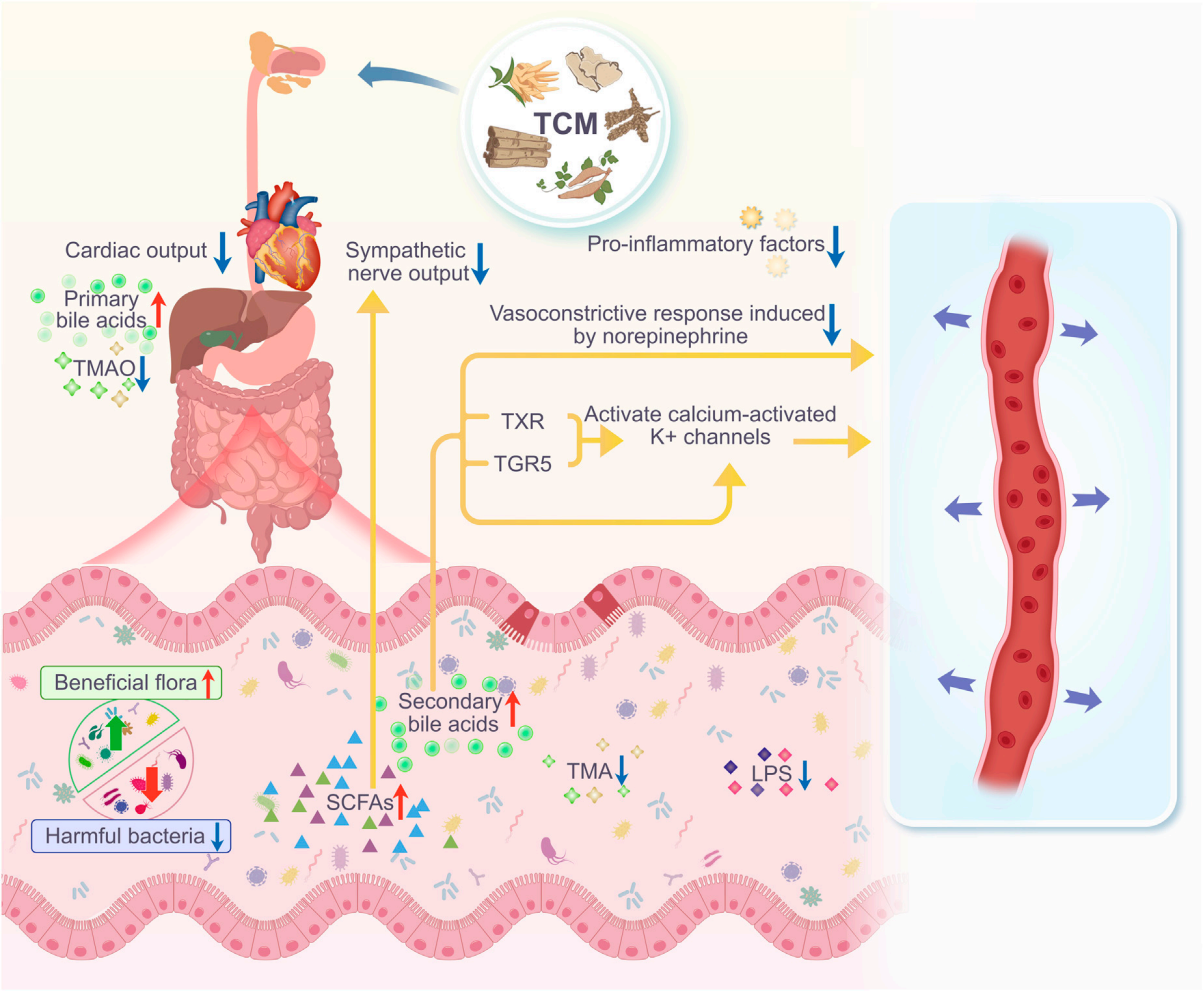
After taking TCM, the diversity and abundance of gut microbiota in hypertensive individuals/animals increase. The number of harmful bacteria decreases, while the number of beneficial bacteria increases, leading to a significant improvement in intestinal barrier function, and an increase in gut microbiota metabolites such as SCFAs and BAs. SCFAs can reduce inflammatory responses and inhibit sympathetic nerve output, effectively lowering blood pressure. Primary BAs are stored in the gallbladder and released into the intestine during digestion, where gut microbiota convert them into secondary BAs. These secondary BAs can also reduce vasoconstriction caused by norepinephrine, and can promote vasodilation by directly activating BK(Ca) channels, or by activating FXR and TGR5 to increase the activity of large-conductance BK(Ca) channels, thereby lowering blood pressure.

TCM compounding involves the combination of two or more TCMs, following certain compounding principles. Some TCM compounds developed to manage hypertension based on their action on intestinal flora have been effective and have been studied more extensively than monomers, single-flavored TCM, and TCM pairs. For example, Sanoshashinto can increase the number of lactobacilli in the intestines of SHRs, thereby regulating blood pressure (Wu et al., 2020). The Huanglian Jiedu decoction may relieve high blood pressure by increasing the intestinal flora of SHR, reducing and increasing the relative abundance of Firmicutes based on the relative abundance of the probiotic *lactobacillus* (Ma et al., 2020). Xiaochaihu decoction combined with irbesartan is more effective than irbesartan alone in lowering blood pressure. This combination reduces the relative abundance of enterococci, yeasts, and Enterobacteriaceae and increases the relative abundance of probiotic lactobacilli (Wu et al., 2022). Fufang-Zhenzhu-Tiaozhi capsule (FTZ)-treated HFS-fed rats with hypertension showed improved intestinal microbial abundance and diversity and increased abundance of Proteobacteria, Verrucomicrobia, and Fusobacteria compared with the model group. Transplantation with FTZ-modulated gut microbiota decreased blood pressure in HFS-fed rats, highlighting that FTZ modulates the intestinal flora and decreases blood pressure (Chen et al., 2022). The Qiangshu Jiangya formula reduces the F/B ratio in NG-nitro-L-arginine methyl ester hydrochloride (L-NAME)-induced hypertension, increases the abundance of SCFA-producing Ruminococcus, and improves oxidative stress *in vivo* (Huang, 2022). Erxian decoction can decrease the relative abundance of TMAO-related Firmicutes and Ruminococcaceae, improve the metabolism of TMAO and its related precursors in circulation, affect the TXNIP/NLRP3 inflammatory pathway, reduce the inflammatory response, and decrease blood pressure elevation in ovariectomized rats (Hu, 2023). The mechanism of action of Taohong Siwu decoction combined with Dubosiella newyorkensis in the treatment of hypertension is the regulation of intestinal microecology, especially the increase in the beneficial bacteria *Lactobacillus* and Allobaculum, the improvement of serum BA metabolism, and the improvement of vascular endothelial function through this action, leading to the effective control of blood pressure (Liu et al., 2023). The compound Qinggan Yishen Qufeng inhibits pathological changes in the ileum and colon, protects the intestinal barrier structure, and regulates the positive correlation with blood pressure in a mouse model of Ang II-induced hypertension. It achieves this by positively influencing the abundance of specific bacterial groups (Actinobacteria, Acidobacteria, Myxococcales, Bacteroidaceae, g_Bacteroidaceae, and g_Tyzzerella, among others). Conversely, it negatively affects bacterial groups, such as Enterobacteriaceae and Rikenellaceae, including g_Alistipes and g_Rikenellaceae_RC9_gut_group, with other abundant specific bacterial groups and blood pressure-related metabolite (DPAn-6, desmethyldeoxycholic acid, and taurocholic acid) levels, thereby reducing blood pressure (Zhen, 2020). Zhengan Xifeng decoction significantly reduces blood pressure in SHRs, regulates the structure of the intestinal flora, reduces the F/B ratio, increases the number of SCFA-producing bacteria, promotes the conversion of lactic acid to butyric acid in the intestinal tract, reduces the levels of d-lactic acid and diamine oxidase (DAO) in the intestinal tract, and maintains the integrity of the intestinal barrier, thus lowering blood pressure (Yu et al., 2019; Xu et al., 2022). Moreover, Chaigui decoction can increase angiotensin-converting enzyme (ACE) two levels in the plasma and decrease renin levels in renal tissues, thereby decreasing the renin to ACE2 ratio. It also improves the intestinal flora by increasing the abundance of beneficial *Bacteroides* and decreasing the abundance of harmful Clostridia. Additionally, Chaigui decoction reduces systolic and diastolic blood pressure, modulates the renin-angiotensin-aldosterone system, affects serum levels of lysophosphatidylcholine, and may further reduce blood pressure by increasing the abundance of S24-7 Bacteroidia, which is negatively associated with blood pressure regulation; this effect has been validated in a hypertensive rat model (Zhu et al., 2023a; Zhu et al., 2023b). The medicinal and food homologous TCM compounding reduces the F/B ratio, increases the abundance of *Lactobacillus*, and regulates serum metabolites and their related metabolic pathways by modulating the intestinal flora structure of two kidneys and one clip (2K1C) rats with hypertension. This compounding reduced blood pressure in a hypertensive rat model, reduced metabolites and their related metabolic pathways, repaired vascular and organ damage, and exerted comprehensive therapeutic effects on hypertension (Guo et al., 2023). The Jiawei Banxia Baizhu Tianma Decoction (MBTD) regulates the structure of the intestinal microbial community by increasing the level of *Streptococcus* species, decreasing the level of Desulfovibrio desulfuricans and *Vibrio desulfuricans*, increasing the expression of short SCFAs and their receptors GPCR41 and GPCR43, enhancing intestinal barrier function, and decreasing the level of LPS in the serum. MBTD inhibits the vascular TLR4/MyD88 pathway, regulates the balance between NO and ET-1, and improves vascular endothelial function, thus effectively improving blood pressure and lipid metabolism disorders in hypertensive rats (Wu et al., 2024). Tianma-Gouteng granules increase the relative abundance of Desulfovibrio, Lachnoclostridium, and Turicibacter and decrease the relative abundance of Allobaculum and Monoglobus by regulating the balance of the intestinal flora in the hypertensive rat model. It further regulates BA metabolism through the gut–hepatic axis, affects the FXR-FGF15-CYP7A1 signaling pathway, and promotes the synthesis and secretion of BAs to comprehensively regulate blood pressure, thus playing an important role in the treatment of hypertension (Yu et al., 2024). The composition of each TCM compounding is listed in Table 2. TCM compounds show therapeutic effects on hypertension by regulating the balance of intestinal flora, increasing the abundance of beneficial bacteria, decreasing the abundance of harmful bacteria, improving the intestinal barrier function, promoting the production of short-chain fatty acids, regulating the level of metabolites related to blood pressure, and influencing the metabolism of BAs through the intestinal–hepatic axis, which in turn integrally regulates blood pressure (Figure 2).

**TABLE 2:** Composition of TCM compound formula.

Compound prescription of Chinese medicine	Traditional Chinese medicine composition
Sanoshashinto	Rhei rhizoma, Scutellariae radix, Coptidis rhizoma
HuanglianJiedu decoction	Coptis chinensis Franch., Phellodendron chinense Schneid., Scutellaria baicalensis Georgi, Gardenia jasminoides Ellis
Xiaochaihu decoction	Bupleurum chinense DC., Codonopsis pilosula (Franch.) Nannf., Scutellaria baicalensis Georgi, Glycyrrhiza uralensis Fisch., Pinellia ternata (Thunb.) Breit., Zingiber officinale Rosc., Ziziphus jujuba Mill
Fufang-Zhenzhu-Tiaozhi capsule	Citri sarcodactylis fructus, Ligustri lucidi fructus, Salviae miltiorrhizae radix et rhizoma, Notoginseng radix et rhizoma, Coptidis rhizoma, Atractylodis macrocephalae rhizoma, Cirsii japonici herba et radix, Eucommiae cortex
QiangshuJiangya formula	Cyathula officinalis, Ilex hainanensis Merr, Epimedium, Eucommia ulmoides, Pueraria
Erxian decoction	CurculigoorchioidesGaertn., Epimedium brevicornu Maxim., Angelica sinensis (Oliv.) Diels, Morinda officinalis How, Phellodendron chinense Schneid., Anemarrhena asphodeloides Bge
TaohongSiwu decoction	Angelica sinensis (Oliv.) Diels, Ligusticum striatum DC., Rehmannia glutinosa (Gaertn.) DC., Paeonia delavayi Franch. Prunus davidiana (CarriŠre) Franch
Qinggan Yishen Qufeng formula	Prunella vulgaris L., Ligusticum chuanxiong Hort., Bupleurum chinense DC., Coptis chinensis Franch, Notopterygium incisum Ting ex H.T. Chang. Saposhnikovia divaricata (Turcz.) Schischk., Cyathula officinalis Kuan, Polygonatum sibiricum Red., Haliotis diversicolor Reeve, Scutellaria baicalensis Georgi, Taxillaria baicalensis Georgi, Taxillus chinensis (DC.) Danser
Zhengan Xifeng decoction	Achyranthes bidentata Bl., Ruddle, Long Gu, Oyster shell, *Chinemys reevesii* (Gray), Paeonia lactiflora Pall., Scrophularia ningpoensis Hemsl. Asparagus cochinchinensis (Lour.) Merr., Melia toosendan Sieb.et Zucc., Hordeum vulgare L., Artemisia scoparia Waldst.et Kit. Glycyrrhiza uralensis Fisch
Chaigui decoction	Bupleurum chinense DC, Glehnia littoralis Fr. Schmidt ex Miq., Pinellia ternata (Thunb.) Breit., Alisma orientale (Sam.) Juzep., Scutellaria baicalensis Georgi, Zingiber officinale Rosc. baicalensis Georgi, Zingiber officinale Rosc., Angelica sinensis (Oliv.) Diels, Paeonia lactiflora Pall., Poria cocos (Schw.) Wolf, Atractylodes macrocephala Koidz., Ligusticum chuanxiong Hort
Medicine and food homologous; Chinese medicine formula	Puerariae lobatae Radir, Prunellae spica, Eucommiae folium, Chrysanthemi flos, Crataegi fructus, *Apium graveolens*
Jiawei BanxiaBaizhu Tianma decoction	Pinelliaternata (Thunb.) Breit., Atractylodes macrocephala Koidz., Gastrodiaelata Bl., Citrus reticulata Blanco, Uncaria rhynchophylla (Mig.) Mig. ex Havil., Poria cocos (Schw.) Wolf, Alisma orientale (Sam.) Juzep., Glycyrrhiza uralensis Fisch
Tianma-Gouteng granules	Gastrodiaelata Blume, Uncaria rhynchophylla (Miq.) Miq. ex Havil, Haliotis diversicolor Reeve, Gardenia jasminoides Ellis, Scutellaria baicalensis Georgi, Achyranthes bidentata Bl., Eucommia ulmoides Oliv. baicalensis Georgi, Leonurus japonicus Houtt., Taxillus chinensis (DC.) Danser, Poria cocos (Schw.) Wolf., Polygonum multiflorum Thunb

## 4 Conclusion

This study systematically reviewed current research on TCM regulation of gut microbiota to control hypertension, and demonstrated consistent findings. Various TCM approaches impacted blood pressure through multiple mechanisms, primarily in modulating the composition of gut microbiota, enhancing intestinal barrier function, regulating gut-derived metabolites, and suppressing inflammatory responses. First, TCM reshapes the microbiota by increasing beneficial bacteria, such as bifidobacterium and *lactobacillus*, and inhibiting harmful bacteria. Second, TCM reduces intestinal permeability by upregulating tight junction proteins, such as ZO-1 and occludin, thus preventing gut-derived toxins (e.g., LPS) from entering the bloodstream, thereby protecting the vascular endothelium. Additionally, TCM influences the production of gut metabolites, including SCFAs and TMAO. SCFAs contribute to vasodilation, whereas TMAO, associated with hypertension, increases vascular resistance and induces endothelial damage. TCM also reduces the expression of inflammatory factors such as IL-6 and TNF-α, mitigating vascular injury caused by hypertension. Several animal studies have shown that TCM significantly lowers systolic and diastolic blood pressure in hypertensive models, with reductions generally ranging from 10 to 50 mmHg, which is clinically meaningful. However, most existing studies are limited by small sample sizes and there is a lack of large-scale randomized controlled trials. Future multi-center clinical trials are essential to verify TCM’s efficacy and safety, optimize therapeutic protocols, and establish a basis for standardized and personalized applications in hypertension management.
